# Biomechanical Characteristics between Bionic Shoes and Normal Shoes during the Drop-Landing Phase: A Pilot Study

**DOI:** 10.3390/ijerph18063223

**Published:** 2021-03-20

**Authors:** Huiyu Zhou, Chaoyi Chen, Datao Xu, Ukadike Chris Ugbolue, Julien S. Baker, Yaodong Gu

**Affiliations:** 1 Faculty of Sports Science, Ningbo University, Ningbo 315211, China; zhouhuiyu@aliyun.com (H.Z.); chencarol@aliyun.com (C.C.); xudatao3@gmail.com (D.X.); 2 School of Health and Life Sciences, University of the West of Scotland, Scotland G72 0LH, UK; U.Ugbolue@uws.ac.uk; 3 Department of Sport and Physical Education, Hong Kong Baptist University, Hong Kong 999077, China; jsbaker@hkbu.edu.hk

**Keywords:** landing task, bionic shoes, landing injury, statistical parametric mapping

## Abstract

With the development of unstable footwear, more research has focused on the advantages of this type of shoe. This type of shoe could improve the muscle function of the lower limb and prevent injury risks in dynamic situations. Therefore, the purpose of this study was to investigate differences in lower-limb kinetics and kinematics based on single-leg landing (SLL) using normal shoes (NS) and bionic shoes (BS). The study used 15 male subject volunteers (age 23.4 ± 1.14 years, height 177.6 ± 4.83cm, body weight (BW) 73.6 ± 7.02 kg). To ensure the subject standardization of the participants, there were several inclusion criteria used for selection. There were two kinds of experimental shoes used in the landing experiment to detect the change of lower limbs when a landing task was performed. Kinetics and kinematic data were collected during an SLL task, and statistical parametric mapping (SPM) analysis was used to evaluate the differences between NS and BS. We found that the flexion and extension angles of the knee (*p* = 0.004) and hip (*p* = 0.046, *p* = 0.018) joints, and the dorsiflexion and plantarflexion of ankle (*p* = 0.031) moment were significantly different in the sagittal planes. In the frontal plane, the eversion and inversion of the ankle (*p* = 0.016), and the abduction and adduction of knee (*p* = 0.017, *p* = 0.007) angle were found significant differences. In the horizontal plane, the external and internal rotation of hip (*p* = 0.036) and knee (*p* < 0.001, *p* = 0.029) moment were found significant differences, and knee angle (*p* = 0.043) also. According to our results, we conclude that using BS can cause bigger knee and hip flexion than NS. Also, this finding indicates that BS might be considered to reduce lower-limb injury risk during the SLL phase.

## 1. Introduction

In the early stages of development, humans did not use any kind of footwear in their daily activities. There is a record of the earliest footwear use from the United States, which could date back to 8300 years ago [1]. About 30,000 years ago, the gracilization of pedal phalanges was found in certain populations, which from the perspective of biomechanics and anatomy can be indirectly explained by the use of shoes [2]. However, Kenya and Ileret, in their research on fossil footprints, have provided at least three instances of direct evidence that humans walked without any shoes 1.52 million years ago [3,4]. In other places, for example South Africa, Tanzania, and Australia, there is stronger evidence for the earliest human beings walking barefoot [5,6,7,8]. Even in the present day, certain indigenous populations use barefoot walking or running in their daily life [9,10,11,12]. These evidences indicate that barefoot is the most primitive human condition, and maybe shoes were not so important in human development.

Barefoot is an unstable structure, based on this situation, which indirectly presents that barefoot walking might have been needed originally by the human structure. Traditionally, shoes have been used for protecting the foot and providing functional support [13]. Normal footwear that provides stability and support functions for the foot could result in overprotection. Consequences of this overprotection may include influence on, and reduced function of, lower limbs [14,15]. For example, this overprotection could reduce muscle strength, Nigg and Sousa further proved that this overprotection could lead to potential injuries when performing exercise [16,17]. Based on these considerations, many studies started focused on unstable shoes, such as RC (reflex control) shoes and MBT (Masai Barefoot Technology) [18]. Moreover, previous studies have suggested using footwear to help people stimulate their balance, thereby reducing impact and improving the cushioning effect [19,20]. The unstable soles of these shoes aim to stimulate human proprioceptive ability and to try to get more muscles involved in the movement and to better distribute the impact of the movement.

“Barefoot shoes” is the original idea of creating unstable shoes. After years of evolution, the cuticle of the human foot has gradually degraded, so protection of the foot is still quite necessary. However, we also want to restore the most primitive state of human beings. Based on these two necessary factors, we design bionic shoes (BS) by combining the functions of barefoot and shoe protection. BS are shoes customized for individuals based on the shape of the soles of their feet. The soles of the feet are scanned to copy the dimensions of each person’s foot shape exactly. Turbanski et al. and Lohrer et al. have suggested that the less contact area between the sole of the shoe and the ground, the greater the sense of movement stimulation [18,21]. The biggest difference between bionic shoes and other unstable shoes is that their instability is only designed according to the posture or motion state of the human body when walking. However, our bionic shoes combine their advantages and add the barefoot form, which more truly reflect and restore the state of barefoot walking. Recently, more and more research has been focused on the development of this type of shoe [22,23,24,25,26,27], such as minimal footwear, which was an important previous study for footwear development [28,29]. These studies focused on basic walking and running performance, and stance also. What we trying to do is to use BS to help athletes who play specific sports reduce injury, or discover some underlying mechanism that we don’t know about. Most of the damage has been caused as a result of landing from heights, and examples of this include basketball, soccer, and volleyball [30,31,32,33]. Athletes have suffered landing injuries for many years, such as knee and ankle injuries, and anterior cruciate ligament injuries. These types of landing injuries can reduce the performances of athletes and could have a detrimental effect on their athletic careers. As a result, research investigating how to minimize injury risks for athletes and how to improve their performance potential during games is very important [34].

In previous studies, landing techniques have been explored by researchers to help athletes avoid landing injuries. One successful technique previously used that has minimized injury has been attempting to land softly. This technique can modify knee loading and increase knee and hip flexion while reducing impact forces [35,36]. Soft landing has been proven to be an effective method used for double-leg landing [37,38,39]. Yeow et al. suggested that individual landing techniques can impact different magnitudes of kinematics, kinetics, and energetics during landing [40]. Cortes et al. demonstrated that there were significantly lower forces during knee flexion angles during the double-leg landing phase than during the single-leg landing (SLL) phase at initial contact [41]. SLL is one of the most basic techniques used in the sport. Previous studies have shown that SLL seems to be more prone to injury risk than double-leg landing. SLL is more directly related to traumatic knee injuries during sports performance [34]. Donohue et al. and Yeow et al. demonstrated that SLL included greater lower extremity loading when compared to landings using double legs [42,43]. Donohue et al. also suggested that the SLL phase is approximately 20–30 degrees less, compared to the double-leg landing phase at peak knee flexion angles [42].

The specific function of BS has not yet been fully understood, nor has the inner mechanism, including its principle, been discovered, according to previous studies. BS can help a lot in injury prevention, and through this study we can better explore the deeper mechanisms of the lower limbs, and better use BS to help athletes to reduce sports injuries. BS could help people to stimulate more muscles, and in doing so engage their motivation—based on this idea, we would like to use BS to decrease the rate of injuries in landing tasks. Probably, this is an important goal of future shoe development. To our knowledge, there are no studies that have investigated BS when performing landing phases in sport and athletic situations. The objective of this study was to investigate possible differences in the lower-limb kinetics and kinematics based on SLL when using NS and BS. We hypothesized that BS will result in higher knee-joint angles and moments, compared with NS during an SLL phase. We also suggested that the angle of the hip joint may vary more in the frontal plane.

## 2. Materials and Methods

### 2.1. Study Design

For our purpose, the subjects were asked to stand on a platform 40 cm high and perform repeated descending movements. All study information was contained and provided on a consent form that was signed by all participants. The study was approved by the Ethics Committee of Ningbo University (protocol code RAGH 20200106).

### 2.2. Participants

From 10 May to 10 June, 15 Chinese male subjects from Ningbo university volunteered for the study (Age 23.4 ± 1.14 years, height 177.6 ± 4.83 cm, body weight (BW) 73.6 ± 7.02 kg). A power analysis from previous research evaluating lower extremity biomechanics between conditions revealed that to achieve 80% power at an alpha criterion level of 0.05, a minimum of 11 participants were required for comparison [34]. To ensure the subject standardization of the participants, there were several inclusion criteria used for selection. These included: (1) All the participants were young amateur athletes. (2) Every participant engaged in sports three times a week (at least). (3) There had been no prior surgery performed on the lower limbs. (4) There were no injuries of any kind on the lower limbs in the last six months, and there were no medical issues that could impact the experimental results. Prior to experimental data collection, all participants were informed about testing, including the purpose, procedures, requirements, and conditions of the study.

### 2.3. Shoes

**Figure 1:**
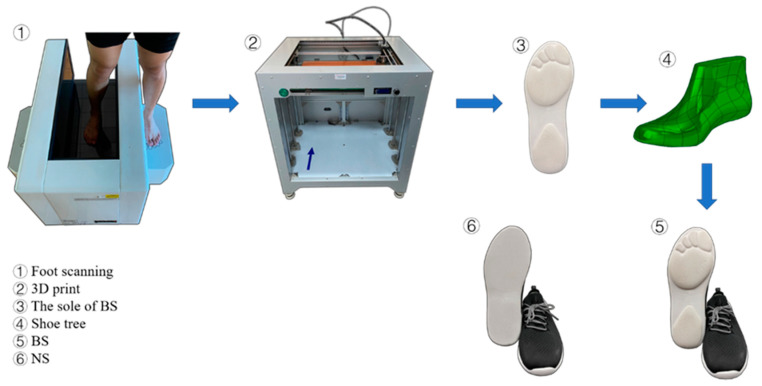
Illustration of shoe-making procedure from initial idea to finished product.

Figure 1 shows that there were two kinds of experimental shoes used in the landing experiment. (1) NS: these test shoes were produced by Ningbo Jiangbei Feibu Sports Goods Co., Ltd. (Ningbo, China). (2) BS: these shoes were customized for each participant based on their individual foot characteristics. A foot-scanning machine was used (VAS-39, Orthobaltic, Lithuania) to scan individual foot shape, then using a 3D print (Dragon(L) 3D Printer, Winbo, China). Based on the data from the foot scanner, a plastic foot model was developed. This scanned data was then given to the shoe factory (Ningbo Jiangbei Feibu Sports Goods Co., Ltd., Ningbo, China), who developed the shoe tree and then manufactured the shoe. The stiffness and materials of both types of shoe were identical [22].

### 2.4. Experiment Protocol

**Figure 2:**
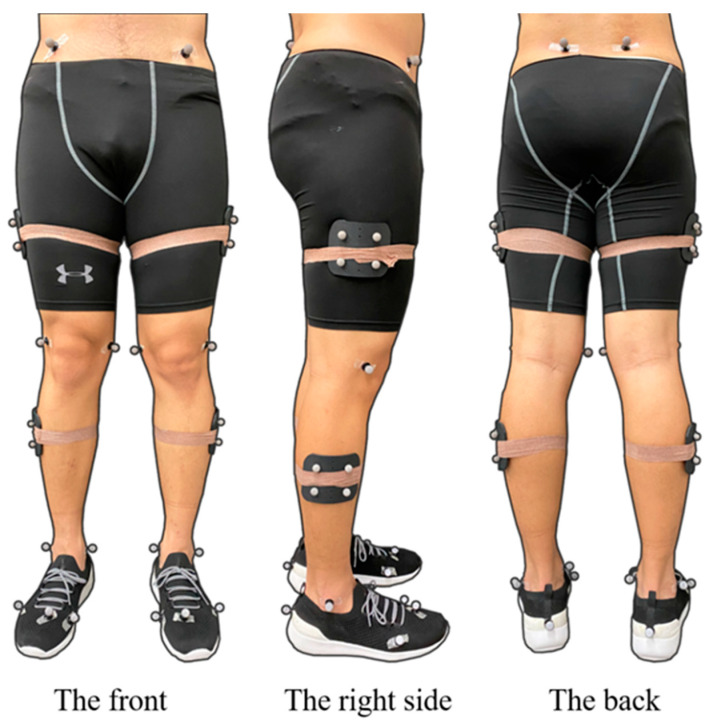
Illustration of marker placements on three sides.

All tests were performed at the Ningbo University Research Academy of Grand Health (Sports Biomechanics Laboratory). A Vicon motion capture system (Oxford Metrics Ltd., Oxford, UK) with eight cameras was used to capture the motion patterns of participants moving during the landing task. The sampling frequency was set at 200 Hz. All participants were required to wear tight shorts and pants. According to previous studies, 20 reflective markers (diameter: 12.5 mm) were secured onto the participants for the identification of movement patterns during the trial. Figure 2 displays the marker placement. The marker locations included: right and left anterior superior iliac spine, left and right posterior superior iliac spine, medial and lateral condyle, medial and lateral malleolus, first and fifth metatarsal heads, distal interphalangeal joint of the second toe. Tracking clusters were placed on the middle and lateral thigh, shank, and right heel [44].

### 2.5. Procedure

**Figure 3:**
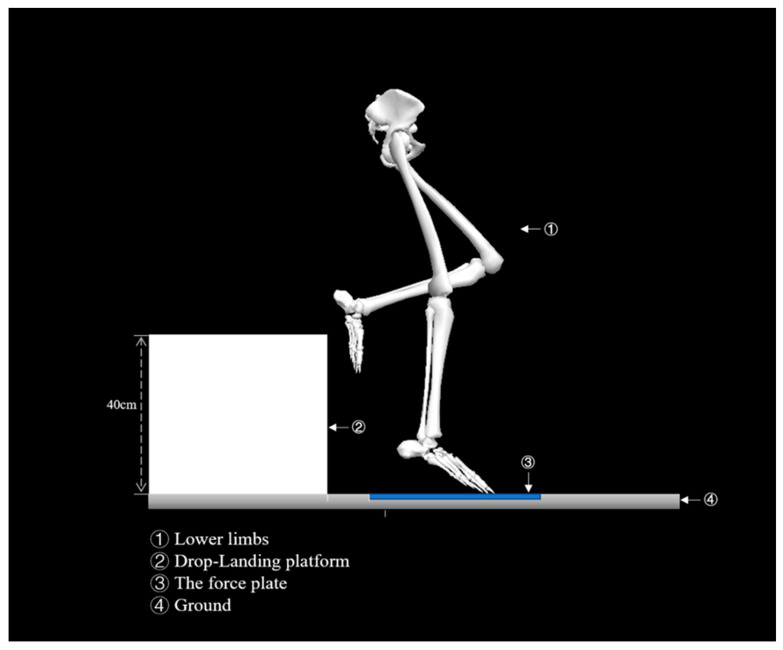
Experimental design for capturing the kinetic and kinematic data during the single-leg landing (SLL) phase for both normal shoes (NS) and bionic shoes (BS).

Participants were required to perform stretching exercises following a warm-up of 10 min at a speed of 8 km/h on the treadmill. Participants wore the same tight shirts and shoes as required for the formal experiment. Familiarization consisted of three practical trials. Further full testing including experimental familiarization was also performed by each participant following warm-up. Figure 3 displays participants positioned themselves 10 cm away from the force plate at a height of 40 cm on a rigid box. A methodology developed for a single-leg drop landing was then used to capture data. Each participant needed to perform the landing task when they heard an audio signal. When they heard the signal, participants jumped onto the force plate, landing on a single leg, using the standardized protocol. Participants were asked to maintain balance on landing for five seconds on the force plate following completion of the full landing phase.

The subjects were asked to choose a shoe at random without knowing which pair of test shoes it was. During the landing task, if participants experienced any kind of loss of balance—for example, the body swinging side to side or trying to touch the ground with the hands, the experiment was recorded as a failure. The dominant leg was used to collect 10 successful data sets, which equated to a total of 20 data sets for each participant using both types of shoes. A 30-s break between each landing task was observed to avoid undue fatigue of participants caused by continuous drop landing. Individual subject fatigue could lead to inaccuracies in data collection.

### 2.6. Data Collection and Processing

This study focused on kinetic and kinematic changes based on different shoes when performing an SLL. Visual 3D (c-motion Inc., Germantown, MD, USA) is customized functional software used to calculate and process kinetic and kinematic variables in the sagittal, frontal, and horizontal planes of the ankle, knee, and hip joint angles and moments using C3D files generated from Vicon Nexus Software. The initial contact point was defined as the vertical surface reaction force exceeding 10N [45]. Data collection was separated into two stages; the first stage was 2 s before initial contact with the ground, the second stage was 3 s after contact with the ground. The description of the frequency of the filter used, was designed in accordance with Winter [46]. The residual analysis of vertical ground reaction force (VGRF) was put into effect in the subsets to determine which was the most appropriate signal-to-noise ratio. The data of VGRF and kinematics were filtered by 10 and 20 Hz fourth-order zero-phase lag Butterworth low-pass filters. The data were imported into MATLAB R2019a (The MathWorks, Teaneck, MA, USA), and an edited code was applied to further analyze the data. The initial ground contact to the maximum knee flexion was defined as the landing phase.

### 2.7. Statistical Analysis

Before statistical analysis, all data were subjected to the Shapiro–Wilk normality test. The Wilcoxon matched-pairs signed-rank test was conducted for non-parametric data if nonconformity was observed. Paired *t*-tests assessed differences in kinetic and kinematic variables between different shoes and SLL.

For SPM analysis, all kinematic and kinetic data of the landing phase were extracted, and the data points were expanded into a time series curve of 101 data points (representing 0–100% of the landing phase) with a custom MATLAB script. Then, the open-source SPM1d script of paired-samples *t*-tests was used for the statistical analysis, and the significance threshold was set at 0.05 [47,48]. All SPM analyses were conducted in MATLAB R2019a using the open-source software package spm1D 0.4 (www.spm1d.org (accessed on 27 November 2019)).

For the traditional discrete variable analysis, a MATLAB script was written to extract the peak VGRF and peak angle points of the knee, hip, and ankle joints in the sagittal, frontal, and transverse planes during the landing stage. All traditional discrete variable analyses were carried out using SPSS 25.0 for Windows™ software (IBM, Armonk, NY, USA). The level of statistical significance was set at *p* <0.05.

## 3. Results

Table 1 and Table 2 show the significant differences between NS and BS at the SLL phase analyzed using paired T-tests for the joints of the lower-limb kinetic and kinematic parameters. Table 3 shows the analysis using the paired *t*-test between NS and BS at the SLL phase on peak VGRF and peak posterior ground reaction force (PGRF). The duration of all the actions in the landing phase is between 323 and 496 ms.

There were significant differences in peak ankle angle inversion (*p* = 0.02), peak hip angle extension (*p* = 0.009), peak knee angle extension (*p* = 0.005), and peak knee abduction (*p* = 0.002) between NS and BS in the kinematics of the lower limb during the SLL phase. For kinetic parameters, peak ankle moment eversion (*p* = 0.048), peak ankle moment inversion (*p* = 0.042), and knee moment external rotation (*p* = 0.006) showed significant differences between NS and BS in the lower limb during the SLL phase.

**Figure 4:**
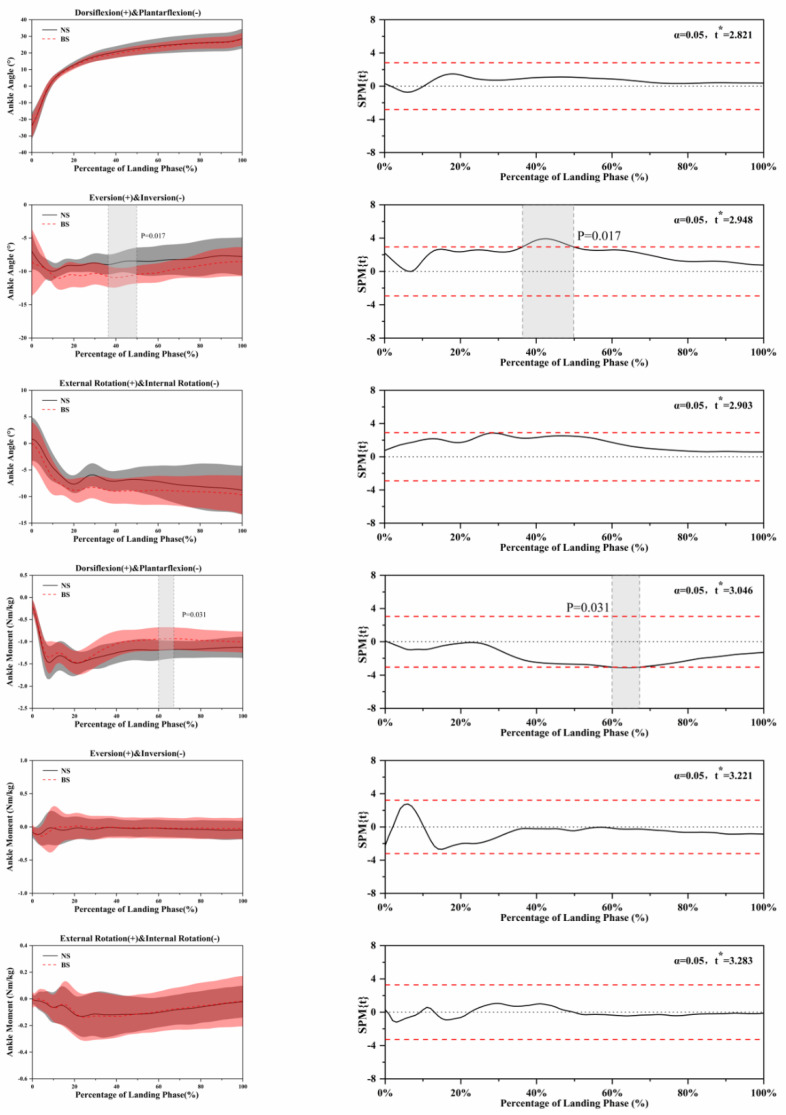
Descriptive results between NS and BS lower-limb statistical parametric mapping results for ankle angles and moments during the SLL phase, *t*-values of the SPM for all participants (post hoc results; dashed red lines represent *p* = 0.05 level). Grey shaded areas display regions that have statistically significant differences. The scale on the left of each image shows the change in the joint angle and the value of *t* *. The scale of 0–100 below each image presents an SLL phase.

**Figure 5:**
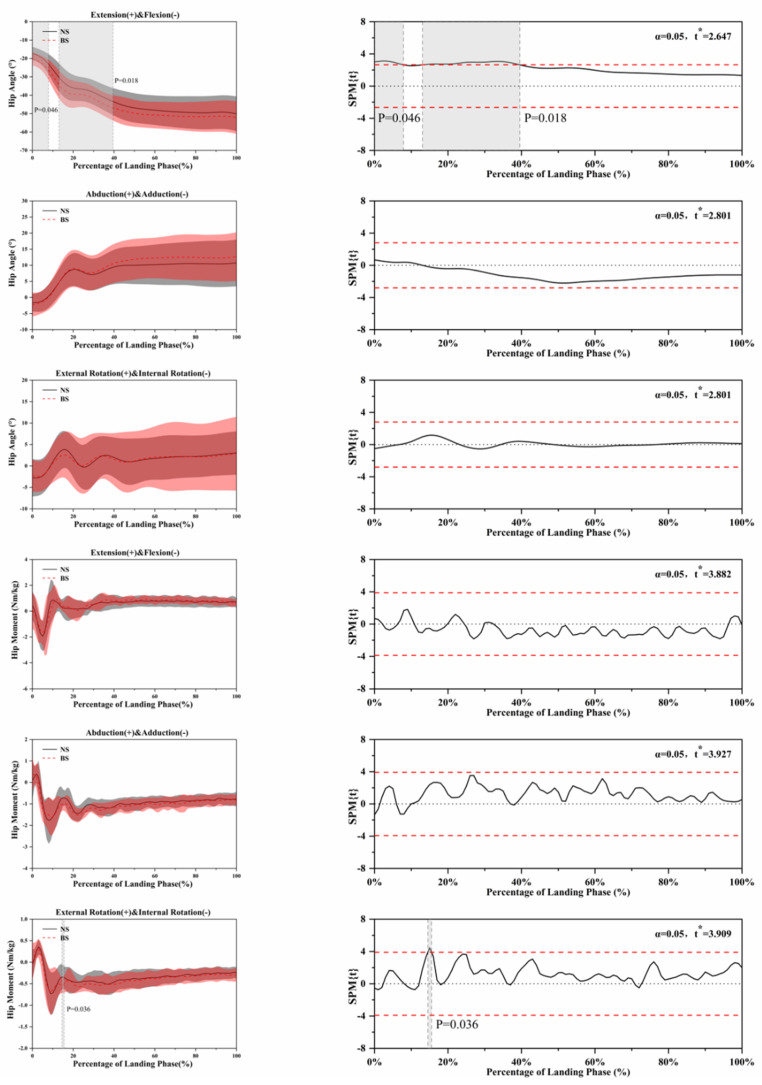
Descriptive results between NS and BS lower-limb statistical parametric mapping results for hip angles and moments during the SLL phase, *t*-values of the SPM for all participants (post hoc results; dashed red lines represent *p* = 0.05 level). Grey shaded areas display regions that have statistically significant differences. The scale on the left of each image shows the change in the joint angle and the value of *t* *. The scale of 0–100 below each image presents an SLL phase.

**Figure 6:**
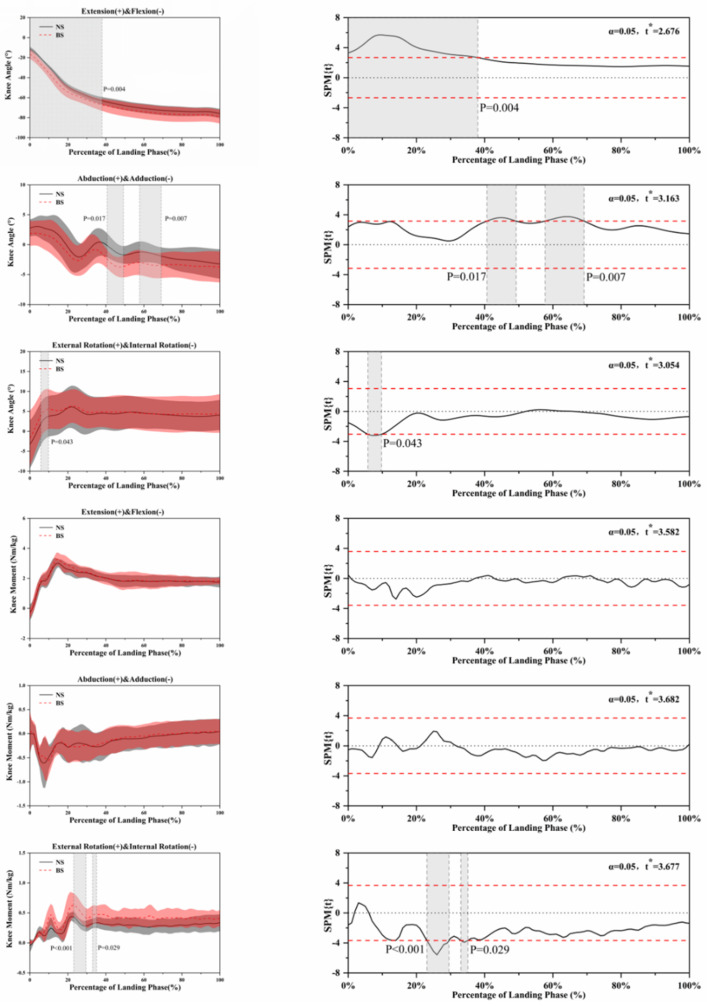
Descriptive results between NS and BS lower-limb statistical parametric mapping results for knee angles and moments during the SLL phase, *t*-values of the SPM for all participants (post hoc results; dashed red lines represent *p* = 0.05 level). Grey shaded areas display regions that have statistically significant differences. The scale on the left of each image shows the change in the joint angle and the value of *t* *. The scale of 0–100 below each image presents an SLL phase.

**Figure 7:**
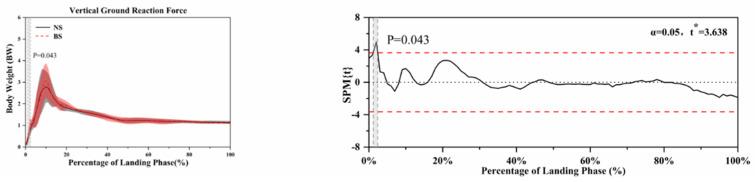
Descriptive results between NS and BS lower-limb statistical parametric mapping results for vertical ground reaction force during the SLL phase, t-values of the SPM for all participants (post hoc results; dashed red lines represent *p* = 0.05 level). Grey shaded areas display regions that have statistically significant differences. The scale on the left of each image shows the change in the joint angle and the value of *t* *. The scale of 0–100 below each image presents an SLL phase.

**Figure 8:**
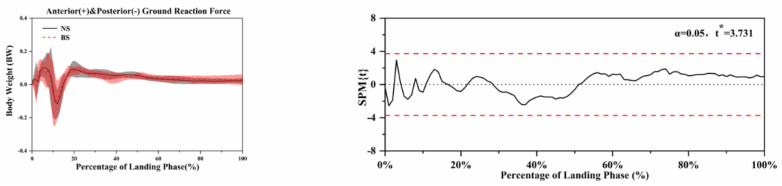
Descriptive results between NS and BS lower-limb statistical parametric mapping results for anterior and posterior ground reaction force during the SLL phase, *t*-values of the SPM for all participants (post hoc results; dashed red lines represent *p* = 0.05 level). The scale on the left of each image shows the change in the joint angle and the value of *t* *. The scale of 0–100 below each image presents an SLL phase.

The SPM analysis using paired *t*-tests in Figure 4, Figure 5, Figure 6, Figure 7 and Figure 8 show the significant differences between the NS and BS during the SLL phase.

Figure 4 shows the kinematic differences using NS and BS in ankle angle and moment. For the ankle angle during eversion and inversion, significant differences (*p* = 0.017) between NS and BS were found during the SLL phase. Further significant differences (*p* = 0.031) were found in the ankle moment during dorsiflexion and plantarflexion between NS and BS during the SLL phase.

Figure 5 shows the kinematic differences using NS and BS in hip angle and moment. Significant differences (*p* = 0.046, *p* = 0.018) were found in hip angle extension and flexion between NS and BS during the SLL phase.

Figure 6 shows the kinematic differences using NS and BS in knee angle and moment. For the knee angle during extension and flexion, significant differences (*p* = 0.004) between NS and BS were found during the SLL phase. Significant differences (*p* = 0.017, *p* = 0.007) were found in the knee angle during abduction and adduction between NS and BS during the SLL phase. Further significant differences (*p* = 0.043) were found in the knee angle during external rotation and internal rotation between NS and BS during the SLL phase. The moment on the horizontal plane is also significant differences (*p* < 0.001, *p* = 0.029) between NS and BS during the SLL phase.

Figure 7 shows the vertical ground reaction force differences using NS and BS, and significant differences (*p* = 0.043) were found between NS and BS during the SLL phase.

**Figure 9:**
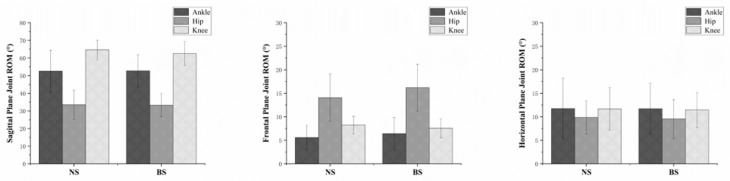
Comparison of the ankle, hip, and knee on the sagittal, frontal, and horizontal plane range of motion during the SLL phase.

Figure 8 shows the anterior and posterior vertical ground reaction force differences using NS and BS. Figure 9 shows the comparison of the ankle, hip, and knee on the sagittal, frontal, and horizontal plane range of motion during the SLL phase. There were no further significant differences found.

## 4. Discussion

The purpose of this study was to investigate any differences between NS and BS when participants performed an SLL. We found that there are significant differences in biomechanical changes between NS and BS during the SLL phase when jumping from a 40 cm height. One of the most significant findings was that the knee and hip flexion angles were significantly greater when the shoes were used than when the shoes were used.

Previous study has proved that termination movements like stop-jumping, side-cutting, and landing could be the main risk for anterior cruciate ligament (ACL) injury [49], and Griffin further suggested that higher trunk, hip, and knee flexion can lead to fewer injuries. Additionally, there were four further studies that indicate that females have stiffer postures during these termination movements [50,51,52,53]; A stiffer landing position will cause greater impact when landing, which will result in greater impact on the knee, thus increasing the risk of knee injury, such as ACL. In our study, BS wearers had higher hip and knee flexion angles in the sagittal plane when performing an SLL, compared with NS wearers. This may be the result of BS activating the body’s protective mechanism and passively increasing trunk flexion angles, and increasing hip and knee flexion angles in the sagittal plane. It is worth mentioning that the previous study suggested that more trunk flexion can reduce injuries. Our results indicated larger trunk flexions, which suggests that the BS can reduce lower-limb injury risks during SLL. It is important to note that although BS can result in higher hip and knee flexion angles in the sagittal plane, there may also be influences on other functions of the lower limbs.

We are unaware of any previous study that has investigated the differences between NS and BS during the SLL phase from 40 cm height; therefore, there might be a limit for comparison to the previous study. However, there are still several studies consistent with our results. The results of Jinkyu, Hossein, and Phillis are in agreement with our knee flexion, hip flexion, and vertical ground reaction force results in a similar drop-landing task [54,55,56]. At the beginning of the landing, when the knee begins to enter the flexion stage, the insertion angle of the patellar tendon with respect to the longitudinal axis of the tibia decreases [57]. Many of the previous researchers have demonstrated that ACL loading could be influenced by the effect of the patellar tendon insertion angle [58,59]. Troy further proved that the greater knee flexion can reduce the impact force on quadriceps/patellar tendon force, which could reduce the force on ACL [60]. According to previous studies, this provides further evidence that the bionic shoe used in this experiment can effectively reduce similar lower-limb injuries to the knee joint.

Regarding BS, there were two studies that may explain why there can be a greater hip and knee flexion angle during the SLL phase. In one study, Nigg investigates the muscle activities of standing phase between NS and unstable shoes including the gastrocnemius, tibialis anterior, biceps femoris, vastus medialis, and for the gluteus medius [16]. However, there is only the tibialis anterior that shows a significant difference. Benno further demonstrates that when the body is in a stable condition, the level of muscle activity required is much lower than when it is in an unstable condition [61]. This suggests that the body does not need to use as many muscles to balance when using regular shoes. Conversely, for example, unstable shoes, the barefoot state, and BS have an unstable condition that could allow more muscles to be passively involved in activities. Therefore, we can conclude that these unstable conditions can actually reduce joint impact force by the high-level activity of muscle.

We combine the barefoot state, which is the original state of humans, and modern footwear to create a new style of shoe. According to our findings, we suggest that BS should be a necessity for future development. BS not only protect the fragility caused by the evolution of the foot, but also present the original status of the foot. If we apply it to basketball, volleyball, and other sports, it may help more athletes avoid lower-limb injury caused by landing, so that the level of human sport can be further advanced. Science and technology change life, and BS use science and technology to protect health. We need to conduct further exploration on the internal mechanisms of BS. We are also open to making joint efforts with researchers in other areas that may help.

It is undeniable that our study has some limitations. Firstly, we recruited only healthy males as our subjects. Females are also an important subject group, and further research is needed to investigate responses in female subjects. Secondly, the hardness of the BS needs further exploration. Differences in sole materials will make a difference to the experimental findings, and we need to investigate further the most suitable hardness for optimal performance and comfort. Thirdly, we looked only at changes in the dominant legs. Further investigations should focus on the changes in the muscular skeletal system using electromyography. Finally, the results were unavoidably influenced by the sample size.

## 5. Conclusions

In summary, this study compared and analyzed NS and BS by quantifying kinetic and kinematic changes during the SLL phase when jumping from a 40 cm height. We found that when using BS in performing an SLL phase, the hip and knee flexion angles have bigger flexion angles than those observed using NS. Also, we compared our findings with previous studies and found that BS might be a great method for reduce lower-limb injury risk during an SLL phase. Further investigations should focus on the changes in muscle using electromyography and expand the sample size to validate our findings.

## Figures and Tables

**Table 1:** Comparison of all kinematic variables between NS and BS during the SLL phase when jumping from 40 cm heights.

Joint Kinematics		NS Mean ± SD	BS Mean ± SD	*p* Value
Ankle Angle	Dorsiflexion	28.62(6.089)	28.35(3.72)	0.774
	Plantarflexion	−23.93(8.33)	−24.4(6.9)	0.763
	Eversion	−5.33(2.49)	−6.14(3.41)	0.257
	Inversion	−10.94(1.12)	−12.56(1.81)	0.020 *
	External Rotation	0.99(4.2)	0.06(4.13)	0.401
	Internal Rotation	−10.75(2.91)	−11.65(2.58)	0.301
Hip Angle	Extension	−17.27(3.76)	−20.17(3.69)	0.009 *
	Flexion	−50.77(9.48)	−53.42(8.58)	0.105
	Abduction	12.18(6.54)	13.66(7.46)	0.200
	Adduction	−1.91(5.03)	−2.54(3.97)	0.481
	External Rotation	5.43(4.73)	5.64(6.85)	0.861
	Internal Rotation	−4.42(3.88)	−3.91(4.1)	0.615
Knee Angle	Extension	−11.17(2.24)	−16.5(5.06)	0.005 *
	Flexion	−75.8(4.74)	−79.02(6.92)	0.129
	Abduction	3.89(2.03)	2.59(2.37)	0.002 *
	Adduction	−4.37(2.28)	−4.97(2.21)	0.179
	External Rotation	8.03(4.55)	8.38(4.76)	0.512
	Internal Rotation	−3.65(5.81)	−3.08(4.79)	0.472

Note: “*” indicates the significant difference of kinematics variables between different shoes at SLL phase from 40 cm heights in the dominant leg (*p* < 0.05). NS: normal shoes; BS: bionic shoes; SD: standard deviation.

**Table 2:** Comparison of all kinetic variables between NS and BS during the SLL phase when jumping from 40 cm heights.

Joint Kinetics		NS Mean ± SD	BS Mean ± SD	*p* Value
Ankle Moment	Dorsiflexion	−0.19(0.16)	−0.19(0.16)	0.933
	Plantarflexion	−1.63(0.31)	−1.6(0.21)	0.767
	Eversion	0.1(0.2)	0.14(0.21)	0.048 *
	Inversion	−0.17(0.14)	−0.21(0.2)	0.042 *
	External Rotation	0.06(0.07)	0.08(0.11)	0.342
	Internal Rotation	−0.18(0.14)	−0.19(0.15)	0.772
Hip Moment	Extension	1.89(0.97)	2.12(0.91)	0.180
	Flexion	−2.57(0.76)	−2.71(0.67)	0.323
	Abduction	0.85(0.52)	0.84(0.59)	0.935
	Adduction	−2.45(0.72)	−2.22(0.54)	0.085
	External Rotation	0.41(0.17)	0.47(0.26)	0.375
	Internal Rotation	−0.92(0.37)	−0.98(0.42)	0.476
Knee Moment	Extension	3.24(0.3)	3.43(0.43)	0.162
	Flexion	−0.28(0.53)	−0.35(0.35)	0.690
	Abduction	0.32(0.23)	0.3(0.28)	0.731
	Adduction	0.89(0.39)	−0.85(0.27)	0.646
	External Rotation	0.5(0.08)	0.69(0.2)	0.006 *
	Internal Rotation	−0.07(0.11)	−0.01(0.03)	0.089

Note: “*” indicates the significant difference of kinetics variables between different shoes at SLL phase from 40 cm heights in the dominant leg (*p* < 0.05). NS: normal shoes; BS: bionic shoes; SD: standard deviation.

**Table 3:** Comparison of all kinetic variables between NS and BS during the SLL phase when jumping from 40 cm heights.

Ground Reaction Force	NS Mean±SD	BS Mean±SD	*p* Value
Peak VGRF	3.55(0.34)	3.64(0.36)	0.332
Peak PGRF	−0.19(0.08)	−0.18(0.13)	0.878

Note: NS: normal shoes; BS: bionic shoes; SD: standard deviation; VGRF: vertical ground reaction force; PGRF: posterior ground reaction force.

## Data Availability

Datasets for the current study are available from the corresponding author upon reasonable request.
